# Using CF11 cellulose columns to inexpensively and effectively remove human DNA from *Plasmodium falciparum*-infected whole blood samples

**DOI:** 10.1186/1475-2875-11-41

**Published:** 2012-02-10

**Authors:** Meera Venkatesan, Chanaki Amaratunga, Susana Campino, Sarah Auburn, Oliver Koch, Pharath Lim, Sambunny Uk, Duong Socheat, Dominic P Kwiatkowski, Rick M Fairhurst, Christopher V Plowe

**Affiliations:** 1 Howard Hughes Medical Institute, University of Maryland School of Medicine, Baltimore, MD, USA; 2 WorldWide Antimalarial Resistance Network Molecular Module, University of Maryland School of Medicine, Baltimore, MD, USA; 3 National Institute of Allergy and Infectious Diseases, National Institutes of Health, Bethesda, MD, USA; 4 Wellcome Trust Sanger Institute, Hinxton, Cambridge, UK; 5 Global Health Division, Menzies School of Health Research, Charles Darwin University, Darwin, Australia; 6 Wellcome Trust Centre for Human Genetics, University of Oxford, Oxford, UK; 7 National Centre for Parasitology, Entomology and Malaria Control, Phnom Penh, Cambodia; 8 Center for Vaccine Development, University of Maryland School of Medicine, Baltimore, MD, USA

**Keywords:** CF11, Cellulose powder, Leukocyte depletion, *Plasmodium falciparum*, Malaria, Next-generation sequencing

## Abstract

**Background:**

Genome and transcriptome studies of *Plasmodium *nucleic acids obtained from parasitized whole blood are greatly improved by depletion of human DNA or enrichment of parasite DNA prior to next-generation sequencing and microarray hybridization. The most effective method currently used is a two-step procedure to deplete leukocytes: centrifugation using density gradient media followed by filtration through expensive, commercially available columns. This method is not easily implemented in field studies that collect hundreds of samples and simultaneously process samples for multiple laboratory analyses. Inexpensive syringes, hand-packed with CF11 cellulose powder, were recently shown to improve *ex vivo *cultivation of *Plasmodium vivax *obtained from parasitized whole blood. This study was undertaken to determine whether CF11 columns could be adapted to isolate *Plasmodium falciparum *DNA from parasitized whole blood and achieve current quantity and purity requirements for Illumina sequencing.

**Methods:**

The CF11 procedure was compared with the current two-step standard of leukocyte depletion using parasitized red blood cells cultured *in vitro *and parasitized blood obtained *ex vivo *from Cambodian patients with malaria. Procedural variations in centrifugation and column size were tested, along with a range of blood volumes and parasite densities.

**Results:**

CF11 filtration reliably produces 500 nanograms of DNA with less than 50% human DNA contamination, which is comparable to that obtained by the two-step method and falls within the current quality control requirements for Illumina sequencing. In addition, a centrifuge-free version of the CF11 filtration method to isolate *P. falciparum *DNA at remote and minimally equipped field sites in malaria-endemic areas was validated.

**Conclusions:**

CF11 filtration is a cost-effective, scalable, one-step approach to remove human DNA from *P. falciparum*-infected whole blood samples.

## Background

Recent technological advances have enabled next-generation genomic and transcriptomic analysis of *Plasmodium falciparum *from parasitized whole blood samples without the need for culturing. High-density genotyping of parasites obtained directly from patients with malaria has improved our understanding of population structure and genomic and transcriptional variation [1,2]. Highly parallel sequencing is currently being used to identify the genetic determinants of clinically relevant phenotypes including drug resistance, vaccine escape and disease severity. Importantly, genomic characterization of parasite populations in patients captures intra-host diversity and prevents the potential loss of sequence data from phenotype-conferring parasite isolates during their culture adaptation.

The performance of highly parallel sequencing platforms, such as Illumina, is greatly enhanced in sample preparations with a high parasite-to-human nucleic acid ratio [3]. Such high ratios can be achieved by either selectively capturing parasite nucleic acids or by removing human material from the sample. Hybrid selection [4] using RNA 'baits' complementary to the *P. falciparum *genome can achieve over 40-fold enrichment of parasite DNA and can be performed at any time following DNA extraction [5]; however, at $250 USD per sample, this technique may be prohibitively costly for epidemiological or population-level studies.

The alternative, leukocyte depletion, must be performed in field sites soon after blood collection and before transport and storage. Commercially available magnetic columns have been used to rapidly isolate parasitized red blood cells (RBCs) from uninfected RBCs and leukocytes [6]. However, magnetic purification depends on short-term culturing of patient blood to transform ring-stage parasites to haemozoin-rich trophozoites and schizonts, which requires equipment for *in vitro *parasite cultivation. Furthermore, parasites obtained directly from patients mature at different rates in culture, resulting in inconvenient time-points for purification. The current standard for leukocyte depletion of parasitized blood for *P. falciparum *nucleotide sequencing is a two-step process: centrifugation using Lymphoprep or another sucrose density gradient solution, followed by filtration using Plasmodipur filters [3]. This 'LP' method is effective but difficult to scale-up in field settings because it requires extensive handling and transfer of blood, training to perform sensitive steps, and costly commercial reagents and materials.

Filtration with hand-made columns has been used as an inexpensive and less time-consuming alternative for leukocyte depletion of *Plasmodium*-infected blood for decades [7,8]. Recently, non-woven fabric filters [9] and plastic syringes filled with CF11 cellulose powder (Whatman) [10] were shown to effectively remove leukocytes and platelets from *Plasmodium vivax*-infected blood, where their phagocytic and degranulating activities may interfere with some *ex vivo *studies. CF11 filtration has also been used to improve microarray-based transcriptome analysis of *P. vivax-*infected blood [2,11]. CF11 cellulose is thought to work by trapping leukocytes by size exclusion and/or interactions between cellulose hydroxyl groups and leukocyte surface molecules. In this study, laboratory-adapted *P. falciparum *clones were used to adapt CF11 columns to remove human DNA from *P. falciparum*-infected blood for genomic studies, compare CF11 filtration to the established LP method, and validate a centrifuge-free option for CF11 filtration. Both methods were also compared in a field setting using *P. falciparum *isolates obtained directly from patients with malaria. Furthermore, routine CF11 filtration of parasitized blood collected from patients in a second field site with minimal facilities was successfully implemented.

## Methods

### Blood samples

Experiments using laboratory-adapted *P. falciparum *clones were conducted at the University of Maryland, Baltimore. Blood obtained from healthy volunteers was mixed with purified *P. falciparum *parasites (3D7 or Dd2 clones) to achieve parasite densities of 10,000/μl and 5,000/μl. These samples were stored at 4°C for up to two hours prior to CF11 or LP filtration.

Experiments using non-adapted *P. falciparum *isolates were conducted in August 2010 at Sampov Meas Referral Hospital, Pursat, Cambodia. Blood samples for these experiments were collected from patients with falciparum malaria who were ≥ 10 years old and had a wide range of parasite densities (≥ 10,000/μl). These samples were held at 4°C for up to six hours prior to CF11 or LP filtration. Additional blood samples were collected in November to December 2010 at Lumphat District Health Centre, Ratanakiri, Cambodia. Blood samples were obtained from patients with falciparum malaria who were ≥ 1 year old and had a wide range of parasite densities ≥ 10,000/μl. These samples were held at 4°C for up to six hours prior to CF11 filtration. Following CF11 filtration, samples were stored on ice for up to six hours and transported on ice for up to 12 hours to Phnom Penh for centrifugation and RBC pellet storage.

All blood samples were collected in EDTA or CPDA-1 tubes. Heparin was not used as an anticoagulant as it thought to inhibit *Taq *polymerase during DNA amplification.

Blood samples from healthy volunteers were collected under a protocol approved by the Institutional Review Board of the University of Maryland School of Medicine. Blood samples from patients with falciparum malaria were collected under protocols approved by the Institutional Review Board of the National Institute for Allergy and Infectious Diseases and the Cambodian National Ethics Committee for Health Research (http://ClinicalTrials.gov identifiers: NCT00341003 and NCT01240603). Written, informed consent was obtained from all study participants or their parents or guardians.

### Leukocyte depletion

**Figure 1:**
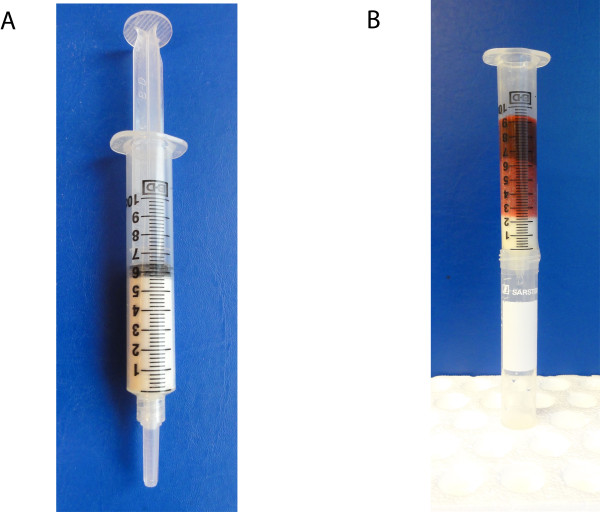
**Packed CF11 column ready for storage, shipping or use (A) and filtration of blood-PBS mixture through CF11 column into collection tube (B)**.

Lymphoprep (Axis-shield, Oslo, Norway) + Plasmodipur (Euro-diagnostica, Arnhem, Netherlands) filtration ('LP') was performed as previously described [3]. CF11 columns were loosely filled to the 10-ml mark, packed to the 5.5-ml mark with CF11 cellulose powder (Whatman, Kent, UK), and wetted with isotonic phosphate buffered saline (PBS), as described [10]. Initial tests indicated that plasma separation prior to CF11 filtration of blood did not affect human DNA depletion and that washing with PBS after CF11 filtration improved recovery of parasite DNA. CF11 filtration was therefore carried out as follows. Parasitized blood or blood/parasite mixtures were diluted with an equal volume of PBS. These samples were added to the CF11 column and allowed to flow through by gravity. To wash the sample, 5 ml PBS was added to the column and allowed to pass through by gravity. A plunger was then inserted into the top of the column and the final few drops pushed through the column. The filtrates were centrifuged for 10 minutes at 1,000 *x *g and the supernatants removed. RBC pellets from all filtrates in all experiments were stored at -20°C until DNA extraction. A packed CF11 column and filtration of blood diluted with PBS are shown in Figures 1A and 1B. The full CF11 filtration procedure is available on the WWARN website [12].

### Experimental design

#### CF11 versus LP filtration, without modifications

To compare the CF11 and LP methods for leukocyte depletion in a laboratory setting, three replicates of blood-parasite mixtures (3-ml volume; 10,000 parasites/μl) were prepared and filtered in parallel using each method

### Experimental design

#### CF11 versus LP filtration, without modifications

To compare the CF11 and LP methods for leukocyte depletion in a laboratory setting, three replicates of blood-parasite mixtures (3-ml volume; 10,000 parasites/μl) were prepared and filtered in parallel using each method. Unfiltered blood-parasite mixtures were prepared as control samples. To compare the two methods in a field setting (Pursat, Cambodia), 8 ml blood from 15 patients with falciparum malaria were obtained and split into two 4-ml aliquots. Each aliquot was processed by either the CF11 or LP method.

#### CF11 filtration, with procedural modifications

To test a centrifuge-free method of CF11 filtration, three replicates of blood-parasite mixtures (3-ml volume; 10,000 parasites/μl) were prepared, filtered through CF11 columns, and held overnight at 4°C to allow RBCs pellet by gravity. Supernatants were removed the following day. To investigate the effect of decreased CF11 column length on leukocyte depletion, three replicates of blood-parasite mixtures (3-ml volume; 10,000 parasites/μl) were filtered through columns loosely filled with CF11 to the 8-ml mark and packed to the 4-ml mark.

#### Modifications in blood volume and parasite density

To investigate the effect of low sample volume on CF11 filtration, three replicates of 1.5-ml blood-parasite mixtures containing 10,000 parasites/μl were prepared and filtered in parallel through CF11 columns loosely filled to the 5-ml mark and packed to the 2.5-ml mark. To investigate the effect of low parasite density on CF11 filtration, three replicates of 3-ml blood-parasite mixtures containing 5,000 parasites/μl were prepared and filtered in parallel through CF11 columns packed to the 5.5-ml mark. In both experiments, unfiltered blood-parasite mixtures were prepared as control samples. The effect of parasite density on CF11 and LP filtration was tested in Pursat, Cambodia, using *P. falciparum *isolates obtained from patients with malaria who had parasite densities ranging from 20,000-475,000/μl.

#### Routine blood collection for further validation and sequencing

To determine how CF11 filtration performed during a routine blood collection protocol, 3 ml blood were obtained from 51 patients presenting with falciparum malaria in Ratanakiri, Cambodia. Blood samples were passed through CF11 columns and the filtrates were transported on ice to the laboratory in Phnom Penh, where the samples were centrifuged to obtain RBC pellets.

### DNA quantification

DNA was extracted from leukocyte-depleted and unfiltered control samples using Qiamp DNA Blood Midi Kits (Qiagen, Venlo, Netherlands) according to the manufacturer's protocol. Total DNA from experiments using *P. falciparum *clones was estimated with a SpectraMax M2 Microplate Reader (Molecular Devices, Sunnyvale, CA, USA) using the Quant-IT PicoGreen dsDNA Assay Kit (Invitrogen, Carlsbad, CA, USA). The standard curve ranged from 0.20 to 50 ng/μl. Total DNA from experiments using *P. falciparum *isolates was estimated with a Qubit 2.0 Fluorometer (Invitrogen, Carlsbad, CA, USA) using both the dsDNA HS and BR Assay kits.

Quantitative real-time PCR (qPCR) was used to measure the relative amounts of human and parasite DNA in each sample. Primers targeting the human LRAP (F: 5'-ACGTTGGATGAATTTTCCACTGGATTCCAT-'3; R: 5'-ACGTTGGATGTGAACCATGCTCCTTGCATC-'3) and TLR9 (F: 5'-ACGTTGGATGCAAAGGGCTGGCTGTTGTAG-'3; R: 5'- ACGTTGGATGTCTACCACGAGCACTCATTC-'3) genes and the *P. falciparum *AMA-1 gene (F: 5'-ACGTTGGATGGATTCTCTTTCGATTTCTTTC-'3; R: 5'-ACGTTGGATGTGCTACTACTGCTTTGTCCC-'3) were used to amplify DNA from samples, negative controls and standards in duplicate. Amplification occurred in an Applied Biosystems 7300 or StepOne real-time PCR machine. Human placental and *P. falciparum *(3D7 clone) genomic DNA standards ranged from 0.20 to 50 ng/μl. For each 25 μl reaction, 12.5 μl SYBR Green qPCR Master Mix (Applied Biosystems, Foster City, CA, USA) was mixed with 1.5 μl of each forward and reverse primers at 2 μM concentrations, 7.5-8.5 μl water, and 1-2 μl of template DNA. Reaction conditions were previously reported [3]: an initial denaturing step of 95°C for 10 minutes, five cycles of 94°C for 45 seconds, 56°C for 45 seconds, 72°C for 45 seconds, followed by 30 cycles of 94°C for 45 seconds, 65°C for 45 seconds and 72°C for 45 seconds and one dissociation cycle (conditions vary by real-time PCR instrument). Results were analysed using Applied Biosystems 7300 System SDS or StepOne v2.0 software.

qPCR quantification of AMA-1 and TLR9 or LRAP was used to determine the proportions of *P. falciparum *and human DNA in the sample. Total DNA was estimated by multiplying the concentration given by Spectramax or Qubit by the extracted sample volume.

### Statistical comparisons

Means, variance, standard errors and statistical comparisons of means were calculated using Stata 11 software. Boxplots indicating medians, interquartile ranges and outliers were also generated using Stata 11.

## Results

### Comparison of unmodified LP and CF11 filtration for leukocyte depletion

**Figure 2:**
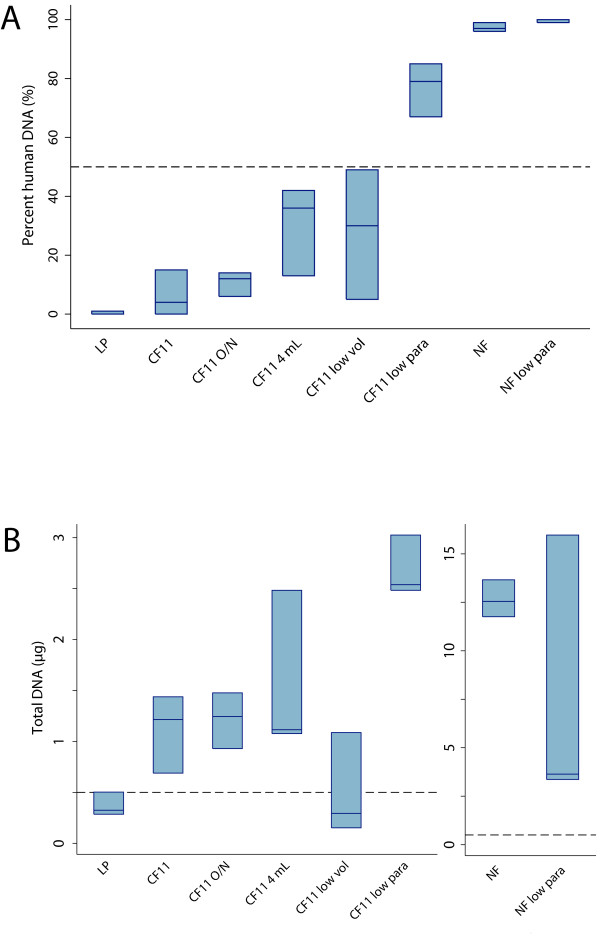
**Percent human DNA (A) and total DNA (B) as estimated by qPCR after leukocyte depletion of blood-parasite mixtures**. The volume and parasite density of blood-parasite mixtures were 3 ml and 10,000/μl except where stated otherwise. Three replicates for each filtration method were performed. Dashed lines indicate current criteria for Illumina sequencing: < 50% human DNA and > 500 ng total DNA. LP = Lymphoprep + Plasmodipur, CF11 = filtration using a 5.5-ml CF11 column, CF11 O/N = CF11 filtrate held overnight prior to removal of supernatant, CF11 4 ml = filtration using a 4-ml CF11 column, CF11 low vol = filtration of a 1.5-ml blood-parasite mixture containing 10,000 parasites/μl, CF11 low para = filtration of a 3-ml blood-parasite mixture containing 5,000 parasites/μl, NF = no filtration, NF low para = no filtration of a 3-ml blood-parasite mixture containing 5,000 parasites/μl. Box plots show median and interquartile range.

**Figure 3:**
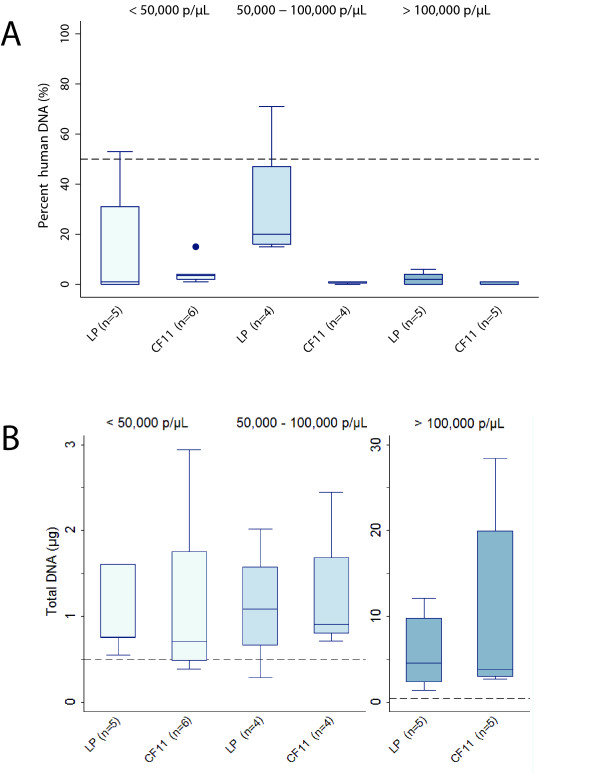
**Percent human DNA (A) and total DNA (B) as estimated by qPCR after leukocyte depletion of *Plasmodium falciparum*-infected blood samples that vary in parasite density**. Light gray shading indicates samples with < 50,000 parasites/μl, medium gray shading indicates samples with 50,000-100,000 parasites/μl, and dark gray shading indicates samples with parasite density > 100,000 parasites/μl. All sample volumes were 4 ml. n = sample size for each method within each range of parasite density. Dashed lines indicate current criteria for Illumina sequencing: < 50% human DNA and > 500 ng total DNA. LP = Lymphoprep + Plasmodipur, CF11 = filtration using a 5.5-ml CF11 column. Box plots show median and interquartile range. Whiskers span the range of data points within 1.5 times the interquartile range of the lower and upper quartiles. Outliers are shown as points.

Filtration of blood-parasite mixtures using unmodified LP and CF11 methods in parallel successfully depleted human DNA to 1% and 6% of total DNA, respectively (Figure 2A), well below the current Illumina sequencing threshold of < 50%. CF11 was more effective than LP in recovering total DNA from blood-parasite mixtures (mean 1.11 μg *vs*. 0.37 μg; p = 0.03) (Figure 2B). All 15 CF11-filtered parasitized blood samples and 12 of 14 LP-filtered parasitized blood samples yielded < 50% human DNA contamination (Figure 3A). Human DNA contamination was lower for parasitized blood filtered by CF11 than by LP (mean 2.4% *vs*. 15.9%; p = 0.03) (Figure 3B). Recovery of total DNA was comparably high in both sets of samples (mean 4.64 μg for CF11 *vs*. 2.86 μg for LP; p = 0.46) One LP-filtered sample failed to produce a human DNA estimate by qPCR and was omitted from the analysis.

### Procedural modifications of CF11 filtration method

To explore whether CF11 filtrates could be processed without centrifugation, CF11-filtered blood-parasite mixtures were refrigerated overnight. The RBC pellet formed during overnight settling was not as tightly packed as that formed during centrifugation and required careful handling when removing supernatant to prevent disruption. This modification resulted in adequate human DNA depletion (mean contamination 11%) (Figure 2A) and total DNA recovery (mean 1.22 μg) (Figure 2B). Columns with less CF11 powder, packed to the 4-ml mark rather than to the 5.5-ml mark, also yielded high total DNA (mean 1.56 μg) and a sufficiently low proportion of human DNA (mean 30%) (Figure 2A and 2B).

### Blood volume and parasite density

All CF11-filtered blood-parasite mixtures (3 ml volume, 10,000/μl parasite density) consistently yielded samples with < 30% human DNA contamination and > 500 ng total DNA (Figure 2A and 2B). However, the *ex vivo *study of *P. falciparum *isolates with interesting phenotypes can be difficult if blood volumes are limited and parasite densities are low. To determine whether such limitations might significantly reduce the quality of DNA samples, CF11 filtrations were repeated using blood-parasite mixtures of lower volume (1.5 ml) or lower parasite densities (5,000/μl). CF11 filtration of low-volume samples (1.5 ml; 10,000 parasites/μl) produced acceptable human DNA contamination (mean 28%) and mean total DNA (0.51 μg), but individual yields of total DNA were highly variable, ranging from 0.15 to 1.09 μg (Figure 2A and 2B). CF11 filtration of low parasite density samples (3 ml; 5,000 parasites/μl) produced adequate amounts of total DNA (mean 2.68 μg) but unacceptable human DNA contamination (mean 77%) (Figure 2A and 2B). Thus, reducing sample volumes or parasite densities by half failed to reliably produce DNA samples acceptable for Illumina sequencing.

Filtration of *P. falciparum*-infected blood obtained from patients with malaria (Pursat, Cambodia) using the CF11 and LP methods consistently achieved high-quality results at parasite densities ranging from 20,000 to 475,000/μl (Figure 3A and 3B). At parasite densities < 50,000/μl and 50,000-100,000/μl, both filtration methods depleted human DNA contamination to ≤ 30% and produced mean DNA yields > 1 μg. Samples with > 100,000 parasites/μl yielded several micrograms of DNA with < 5% human DNA contamination.

### Validation of routine blood collection for illumina sequencing

The 51 parasitized blood samples that were collected and CF11-filtered in Ratanakiri, Cambodia, ranged in parasite density from 15,000 to 290,000/μl and yielded a mean 0.64 μg total DNA with 34% human DNA contamination. All 51 samples met quality standards and have been successfully sequenced on the Illumina platform at the Wellcome Trust Sanger Institute (Hinxton, UK).

## Discussion

As next-generation sequencing technologies improve, both costs and stringency of DNA sample quality requirements continue to fall. Between 2009 and 2011, requirements for Illumina sequencing of *P. falciparum *DNA by the Wellcome Trust Sanger Institute have relaxed from 30% to 50% human DNA contamination. Moreover, the possibility of sequencing multiple DNA samples in a single lane of a flow cell ('multiplex sequencing') has decreased costs immensely while yielding ~2 GB of *P. falciparum *genomic sequence data per sample, with further improvements anticipated in coming years. To take advantage of these increasingly accessible tools, researchers need scalable procedures for DNA sample preparation in minimally equipped field sites.

This study shows that CF11 columns can be used to effectively deplete human DNA from parasitized blood and achieve Illumina sequencing requirements for total DNA yield and percent human DNA contamination, using sample volumes and parasite densities comparable to those used in LP filtration experiments [3]. A major advantage of CF11 over LP filtration is cost-effectiveness. CF11 columns cost approximately one USD each, compared to 10-50 USD for the Plasmodipur filter alone. Additionally, CF11 filtration is a very simple one-step procedure that does not require specialized equipment. A centrifugation step is only used to pellet RBCs after CF11 filtration and can be replaced with a convenient overnight period of refrigeration to allow the RBCs to pellet by gravity. These advantages should enable a large share of the malaria research community to produce parasite DNA samples appropriate for highly parallel sequencing and microarray technologies.

Because CF11 columns are hand-made rather than commercially produced, variation in the length of the column is likely to occur. Samples filtered in columns packed with less CF11 powder exhibited higher human DNA contamination but still met the threshold level for sequencing, indicating that some variation in column preparation can be tolerated. Samples with low blood volumes (1.5 ml) or parasite densities (5,000/μl) did not consistently achieve sufficient human DNA depletion or total DNA recovery when filtered with either the CF11 or LP method. Whole-genome amplification (WGA) after DNA isolation [13] could boost the amount of starting material in cases where human DNA removal is satisfactory but total DNA yield is low. WGA may therefore be useful in enabling genomic characterization of CF11-filtered blood samples with low starting volume, such as those collected from very young children and patients with severe malarial anaemia. It may also be useful in processing blood samples with low parasite densities, as is often the case in patients with a recrudescent parasitaemia after anti-malarial treatment and those with high levels of naturally acquired immunity.

Although specific parameters have not been rigorously tested, observations in the field have indicated that CF11 and parasitized blood can be effectively stored and used in a variety of environmental conditions. CF11 kept in an airtight container with regularly replaced desiccant and stored in a cool place can be used for at least three months, even in climates with high heat and humidity (unpublished observation). EDTA-treated blood stored at 4°C for up to 24 hours prior to CF11 filtration was successfully processed for Illumina DNA sequencing (unpublished observation), but for RNA analysis, no more than six hours of refrigeration is suggested (ZB Bozdech, personal communication). Updates to the range of storage conditions will be made to the WWARN protocol [12] as more information becomes available.

## Conclusions

CF11 filtration currently offers the best cost-effective, one-step approach to remove human DNA from *P. falciparum*-infected whole blood samples and can be successfully implemented in large genome-wide sequencing studies of *P. falciparum *isolates from patients with malaria.

## Abbreviations

RBC: Red Blood Cell; LP: Lymphoprep + Plasmodipur; PBS: Phosphate Buffered Saline.

## Competing interests

The authors declare that they have no competing interests.

## Authors' contributions

MV, CA, SC, OK, PL, SA and SU designed the study and collected the data. MV, CA and SC analysed the data. DS, DPK, RMF and CVP provided guidance and coordination for study design, data collection and analysis. MV wrote the first draft of the manuscript. RMF, CA, SC and CVP edited and revised the manuscript. All authors read and approved the final manuscript.
